# Facial Paralysis and Hearing Loss: A Rare Manifestation of Prostate Cancer Metastases

**DOI:** 10.7759/cureus.1073

**Published:** 2017-03-03

**Authors:** Uroosa Ibrahim, Amina Saqib, Farhan Mohammad, Muhammad R Raza, Nikhil Nalluri, Frank Forte

**Affiliations:** 1 Department of Hematology and Oncology, Staten Island University Hospital; 2 Pulmonary/Critical Care, Staten Island University Hospital; 3 Cardiology, Staten Island University Hospital; 4 Department of Internal Medicine, Staten Island University Hospital

**Keywords:** prostate cancer, facial paralysis, dural metastases, adenocarcinoma prostate, hearing loss, whole brain radiation therapy

## Abstract

Dural prostate metastases (DPM) are a rare manifestation of metastatic prostate cancer seen in approximately one to six percent of cases. Presenting symptoms may include signs of elevated intracranial pressure, headache, altered mental status, or cranial nerve palsies. Hearing loss, sensory changes, dysarthria, and dysphagia are rare symptoms in DPM that were present in our patient. We present a case of a 58-year-old male with a known diagnosis of adenocarcinoma of the prostate presenting with symptoms of acute exacerbation of chronic obstructive pulmonary disease (COPD), sub-acute right-sided hearing loss, and right-sided facial paralysis. Over the course of hospitalization, his neurological symptoms worsened and he developed dysarthria, dysphagia, facial numbness, and worsening back pain. He also appeared more withdrawn and lethargic. The symptoms prompted a neurological evaluation and a magnetic resonance imaging (MRI) revealed multiple areas of bone marrow signal abnormality compatible with osseous metastatic disease. There was extensive smooth dural thickening as well as focal nodular thickening, both consistent with dural metastases. The patient was treated with corticosteroids and external beam radiation therapy (EBRT) with improvement in his back pain and facial paralysis. He died two weeks after completing EBRT. Although rare, DPM should be suspected in males over 50 years of age presenting with neurological symptoms. An MRI with gadolinium is most helpful in delineating the presence and extent of dural and calvarial involvement. Corticosteroids and EBRT have been shown to improve neurological function in up to 67% of patients. However, median survival post-radiation remains approximately three months.

## Introduction

Prostate cancer is a leading cause of mortality in men. Although prostate cancer metastases to bone and retro-peritoneal and pelvic lymph nodes are common, intracranial, particularly dural prostate metastases (DPM) are a rare manifestation of the disease [1]. DPM should be considered in males over 60 years of age presenting with signs of increased intracranial pressure such as altered level of consciousness, headache, or cranial nerve palsies. Magnetic resonance imaging (MRI) with gadolinium is most helpful in delineating the extent of disease. Corticosteroids and external beam radiation therapies (EBRT) are the first line treatments to mitigate symptoms [2].

## Case presentation

A 58-year-old male presented to the emergency room with shortness of breath, fever, and productive cough. The patient’s past medical history was pertinent for prostate cancer metastatic to bone and liver, a prior hip replacement secondary to metastatic disease, chronic obstructive pulmonary disease (COPD), coronary artery disease, hypercholesterolemia, and generalized anxiety disorder. The family history was significant for a paternal grandfather and a maternal uncle with lung cancer. The patient smoked three packs of cigarettes per day for over 45 years. The examination findings were significant for bilateral wheezing, a right-sided facial droop with minimal drooling, and pinpoint pupils. According to the patient, he had the facial droop for the past three weeks. The review of systems was positive for recent onset of right-sided hearing loss.

A review of the patient’s past records revealed that seven months prior to presentation, the patient’s prostate-specific antigen (PSA) was elevated at 54.5 ng/ml and a prostate biopsy was positive for adenocarcinoma in three out of six specimens with a Gleason score of 8 (4+4). The patient was started on leuprolide. Over the ensuing weeks, he developed nocturia and lower back pain and right hip pain disabling him to the point of having to use a cane. A computed tomography (CT) scan of the abdomen and pelvis showed a lucent lesion in the right iliac bone extending superior pubic ramus with a pathologic fracture of the anterior acetabulum. Significant retroperitoneal lymphadenopathy was noted with pre-aortic, para-aortic, and right external iliac chain involvement. The prostate gland was irregular and mildly enlarged. A bone scan revealed metastatic disease in the right scapula, left posterior 7th rib, left anterior 4th rib and right anterior 6th rib, right iliac bone and acetabulum, right femoral neck and intertrochanteric area and a faint focus in the left proximal humeral shaft. The patient underwent hip replacement surgery for extensive and debilitating right hip joint involvement.

**Figure 1:**
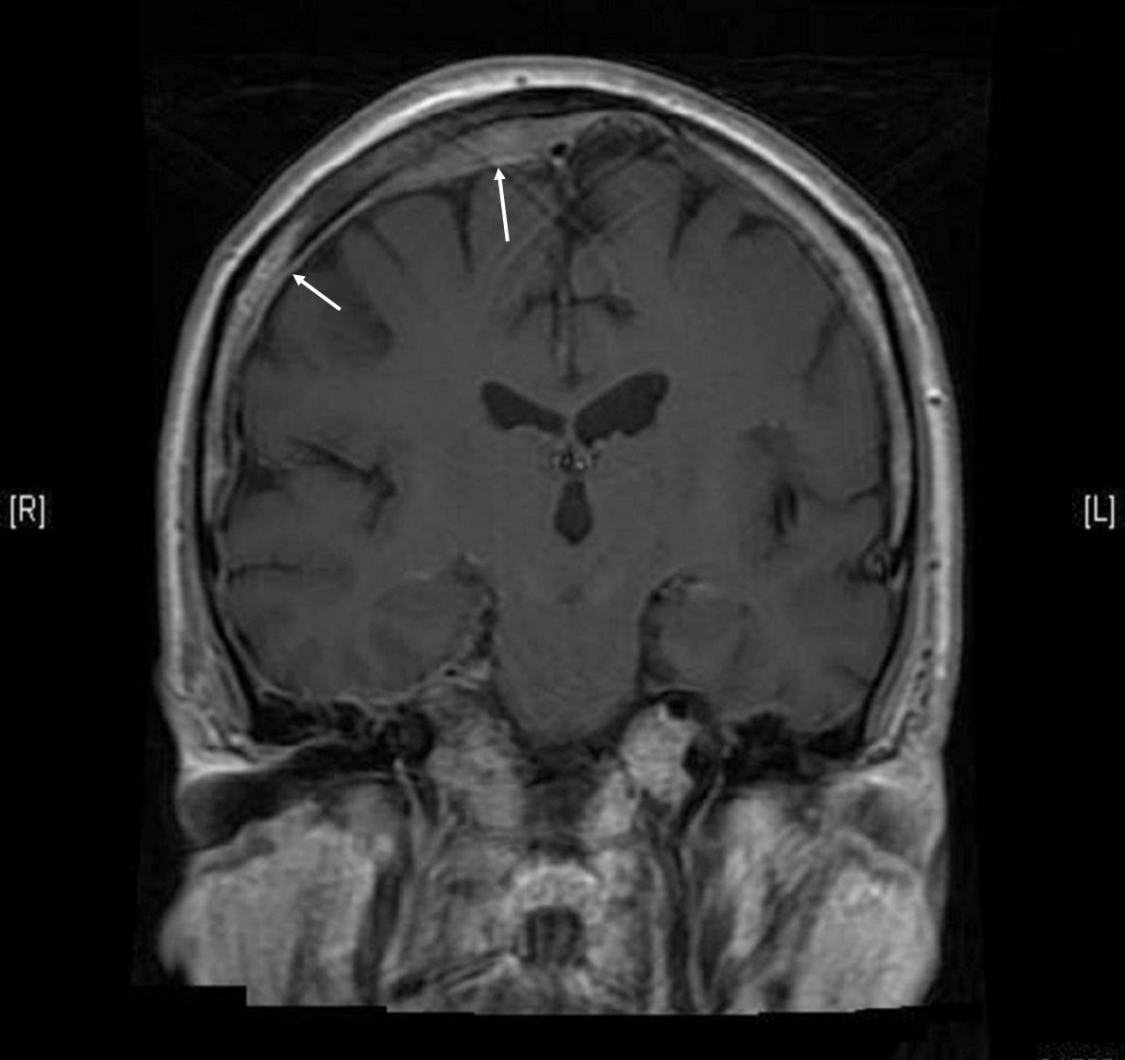
MRI brain Extensive smooth dural involvement over the cerebral hemispheres more prominent on the right. MRI: Magnetic resonance imaging

On our encounter with the patient, he was diagnosed with acute COPD exacerbation and treated with intravenous methylprednisolone, albuterol, and ipratropium nebulization. Over the course of hospitalization, his respiratory symptoms improved but he developed dysphagia, facial numbness, worsening right-sided hearing loss, and back pain. He had also developed dysarthria and appeared withdrawn and lethargic. In view of his symptoms, an MRI brain was done that revealed multiple areas of bone marrow signal abnormality compatible with osseous metastatic disease. An infiltrative mass was noted in the right sphenoid bone expanding the clivus, the right Meckel’s cave, cavernous sinus, petrous apex, and lateral wall of the right orbit. The mass was noted to cause medial displacement of the right lateral rectus muscle and infiltration of the right pterygopalatine fossa, right sphenoid sinus, and pterygoid muscles. The mass was seen to be surrounding the right internal carotid artery. There was extensive smooth dural involvement over the right cerebral and cerebellar hemispheres including the right tentorium, compatible with dural-based metastases [Figure 1]. Areas of focal dural thickening over the high right frontal convexity measuring up to 7 mm and 8 mm were also seen. Special attention was paid to the internal auditory canals given the patient’s complaints of unilateral hearing loss. An abnormal enhancement in the right internal auditory canal was observed likely reflecting extension of dural metastatic disease.

Given the patient’s back pain, an MRI of the spine was also done that revealed T1/T2 hypointense lesions within vertebrae T12, L5, the sacrum and the right iliac bone suggesting sclerotic osseous metastases. Retroperitoneal lymphadenopathy was again noted. Given the above findings, the patient was started on intravenous dexamethasone and EBRT of the dural lesions. Concomitant radiation therapy for lumbar spinal lesions was also started.

The patient was discharged to a rehabilitation facility with the continuation of outpatient radiotherapy. One week later, the patient returned to the hospital after having had an episode of seizures. A routine electroencephalogram (EEG) was negative for any epileptiform activity and the patient was started on levetiracetam. A repeat CT scan revealed no changes from before. His radiation therapy was continued during the hospitalization and he was discharged to the rehabilitation facility. The patient received a total of 30 Gy in 10 fractions to the skull and the lumbosacral spine over four weeks with some improvement in back pain and facial paralysis. It was reported that he died at the nursing home two weeks after completion of radiation therapy.

## Discussion

Dural infiltration is an uncommon finding in extraneural malignancies seen in approximately eight percent of cases [3]. However, when dural lesions are found, the most common primary site is prostate followed by breast, lung, and stomach [4]. DPMs are a rare manifestation of the disease found in approximately one to six percent of cases [1]. About 67% of intracranial metastatic prostate cancer is seen to have dural involvement. Dural metastasis occurs in one to two percent of patients with metastatic prostate cancer and is more common in those with tumors that do not respond to Androgen-deprivation therapy.

Several theories for the mechanism of prostate cancer leptomeningeal metastases exist including direct extension of the tumor from skull deposits, retrograde flow through vertebral veins [5], and bony involvement followed by more distant spread in a chronological order (the cascade theory) [6].

The most common clinical manifestation of DPM is headache. Signs of increased intracranial pressure may be present including altered level of consciousness. Symptoms of dysphagia, dysarthria, diplopia, and facial numbness have also been observed. Cranial nerve palsies may be present depending on the extent of dural infiltration [7]. Our case is unique in terms of presenting with unilateral sub-acute hearing loss and facial paralysis progressing to facial numbness, dysphagia, and dysarthria.

A CT scan is usually done in patients presenting with nervous system findings. However, MRI is more sensitive than CT scan in detecting DPM. Findings on MRI post-gadolinium T1-weighted sequences suggestive of DPM are diffuse smooth dural thickening or nodular thickening in large lesions [8]. Both were present in our patient. On CT scan, these may not be clearly evident and may be misinterpreted as a subdural hematoma. The differential diagnosis in the presence of these findings includes meningioma, lymphoma, tuberculosis, neurosarcoidosis, secondary neoplastic lesions from prostate, breast, lung stomach, or melanoma [9]. In males over 60 years of age with unknown primary and MRI findings suggestive of metastases, the possibility of prostate cancer should be investigated.

EBRT along with corticosteroids is known to partially relieve symptoms of DPM including cranial nerve palsies. EBRT with a total of 40–50 Gy in 20–25 fractions has been recommended as a reasonable treatment for cranial nerve palsies caused by metastases and has been shown to result in neurological improvement in up to 67% of patients. The median survival post-radiotherapy for skull-based lesions is approximately three months [10].

## Conclusions

The neurologic complications of metastatic prostate cancer can be a major cause of morbidity and require early recognition and prompt treatment. Our case emphasizes the need of having a low threshold of diagnosing metastatic disease in a patient with a diagnosis of cancer. Early recognition will aid in avoiding the delay in the management.
